# The H159Y Variant of the BAFF-R Gene (*TNFRSF13C*) Is Unrelated to the Risk of Developing Systemic Lupus Erythematosus and Sjögren’s Disease in a Mexican Population

**DOI:** 10.3390/ijms27020726

**Published:** 2026-01-10

**Authors:** Itzel María Borunda-Calderón, Jazz Alan Corona-Angeles, Noemí Espinoza-García, Miguel Marín-Rosales, Diana Celeste Salazar-Camarena, Edith Oregon-Romero, Ramsés Alejandro Morales-Zambrano, Claudia Azucena Palafox-Sánchez

**Affiliations:** 1 Doctorado en Ciencias Biomédicas, Departamento de Fisiología, Centro Universitario en Ciencias de la Salud, Universidad de Guadalajara, Guadalajara 44340, Jalisco, Mexico; itzel.borunda@alumnos.udg.mx (I.M.B.-C.); jazz.corona@alumnos.udg.mx (J.A.C.-A.); 2 Instituto de Biología Molecular en Medicina y Terapia Génica, Departamento de Biología Molecular, Centro Universitario en Ciencias de la Salud, Universidad de Guadalajara, Guadalajara 44340, Jalisco, Mexico; noemi.espinoza@academicos.udg.mx (N.E.-G.); celeste.salazar@academicos.udg.mx (D.C.S.-C.); 3 Departamento de Reumatología, Hospital General de Occidente, Zapopan 45170, Jalisco, Mexico; miguel.marin@academicos.udg.mx; 4 Instituto de Investigación en Ciencias Biomédicas, Departamento de Clínicas Médicas, Centro Universitario en Ciencias de la Salud, Universidad de Guadalajara, Guadalajara 44340, Jalisco, Mexico; edith.oregon@academicos.udg.mx (E.O.-R.); ramses.morales@academicos.udg.mx (R.A.M.-Z.)

**Keywords:** Systemic Lupus Erythematosus, Sjögren’s Disease, BAFF-R, single-nucleotide polymorphism

## Abstract

Systemic Lupus Erythematosus (SLE) and primary Sjögren’s Disease (SjD) are autoimmune diseases characterized by the presence of autoantibodies that lead to damage in healthy tissues. The production of autoantibodies requires the activation and differentiation of B-lymphocytes into plasma cells. To achieve this effect, BAFF (B-lymphocyte activating factor), APRIL (A proliferation-inducing ligand), and their receptors are key factors. BAFF is a cytokine recognized by BAFF-R (BAFF receptor), which is increased and related to disease activity in both SLE and SjD patients. The H159Y mutation (rs61756766) in the gene encoding the BAFF-R, *TNFRSF13C* (Tumor Necrosis Factor Receptor Superfamily) has been shown in vitro to cause receptor hyperactivation via the NF-κB2 pathway. This study evaluated the frequency of this variant in a western Mexican population and its association with the risk of developing SLE and SjD. Genotypes of the *TNFRSF13C* H159Y (rs61756766) variant were determined by PCR-RFLP assay. sBAFF levels were measured by ELISA. The study included 300 SLE patients, 110 SjD patients, and 300 healthy subjects (HS). HS were in Hardy–Weinberg equilibrium. The data distribution was assessed using the Kolmogorov–Smirnov test. Group comparisons were conducted using the Chi-square test, Fisher’s exact test, or the Mann–Whitney U test, as appropriate. A *p*-value of <0.05 was considered statistically significant. In the Mexican population, allelic and genotypic distribution frequencies of the H159Y variant (rs61756766) were similar between SLE patients and HSs, while the variant was not found in SjD patients. SLE patients carrying the heterozygous CT genotype showed a trend toward higher soluble BAFF (sBAFF) levels than wild-type genotype patients. This variant does not confer risk to SLE or SjD in the Mexican population. However, the heterozygous genotype may be associated with high levels of sBAFF in SLE patients.

## 1. Introduction

Systemic Lupus Erythematosus (SLE) is a chronic autoimmune multisystem disease characterized by the production of autoantibodies, the deposition of immunocomplexes in tissues, and organ dysfunction. Similarly, Sjögren’s Disease (SjD) is another systemic autoimmune condition known for its tendency to induce inflammation in exocrine glands, particularly the salivary and lacrimal glands. This inflammation leads to classic sicca symptoms and extra-glandular manifestations. SLE and SjD have a multifactorial etiology; however, environmental factors, genetic modifications, an interferon signature, and autoreactive B and T cells converge to lead to a loss of immune tolerance and an aberrant immune response in both diseases [1,2,3].

The main pathogenic mechanism in both diseases is the production of autoantibodies that mediate tissue damage; thus, autoreactive B-cell clones play a significant role during the progression and severity of the disease. B-lymphocyte activating factor (BAFF) is an essential cytokine for B-cell development, especially during their maturation in the transitional stage, and has been described to be involved in T-cell-independent B-cell activation [4,5,6].

BAFF and APRIL (A proliferation-inducing ligand), both of which are cytokines in the TNF family, have been researched in relation to autoimmune diseases such as SLE and SjD. Elevated serum levels of these ligands, especially BAFF, have been linked to the production of autoantibodies and disease activity, particularly in cases of SLE with lupus nephritis [7]. The clinical relevance of BAFF and the development of B-cells include the blockade with anti-BAFF and anti-CD20 monoclonal therapies [7,8,9].

BAFF has specific binding to the BAFF receptor (BAFF-R, also known as BR3), and both BAFF and APRIL can bind to B-cell maturation antigen (BCMA) and transmembrane-activator and CAML-interactor (TACI) receptors. These three receptors belong to the tumor necrosis factor (TNF) receptor superfamily and are mainly expressed in B-cells, playing an essential role in B-cell ontogeny [10].

BAFF-R is a type III transmembrane protein encoded in the *TNFRSF13C* gene, localized in chromosome 22q13.2, and it is highly expressed in spleen, lymph nodes, and transitional B-lymphocytes. BAFF binding to BAFF-R promotes the activation signals primarily through the alternative nuclear factor NF-kB2 pathway, triggering the recruitment and direct binding of multiple TNF receptor-associated factors (TRAFs), including TRAF3 [11]. In addition, BAFF/BAFF-R can activate the phosphatidylinositol 3-kinase (PI3K/Akt) signaling pathway; both signaling pathways contribute to the activation, survival, maturation, and proliferation of B-cells [10,11].

The *TNFRSF13C* gene has a single-nucleotide polymorphism (rs61756766), known as the H159Y variant. This variant consists of a single nucleotide substitution [cytosine to thymine (C > T)] at position 475 of the third exon, resulting in an amino acid change from histidine to tyrosine in the cytoplasmic tail of the receptor adjacent to the binding motif for TRAF3. This change was studied in non-Hodgkin’s lymphoma, revealing positive effects with increased recruitment of TRAF2, TRAF3, and TRAF6, along with NF-kB activity [12]. The rs61756766 polymorphism was studied and associated previously with severe COVID-19 cases and autoimmune disease. Particularly in patients with SjD, this variant results in BAFF-R hyperactivity through the recruitment of TRAFs and overexpression of NF-kB2 [13,14,15].

Considering the relationship between the BAFF system and the pathophysiology of SLE and SjD, the association of disease severity with BAFF levels, and the increase in the BAFF-R activity in the presence of the H159Y variant, this study aims to associate the *TNFRSF13C* gene variant with susceptibility to develop SLE and SjD in the Mexican population.

## 2. Results

### 2.1. Clinical and Demographic Features in Study Groups

A total of 300 patients with SLE, 110 with SjD, and 300 HS were included in the study. Female participants predominated across all groups [SLE: 274 (91%), SjD: 110 (100%), and HS: 237 (91.1%)]. The median age was 34 years (IQR: 25–37) for SLE patients, 56 years (IQR: 48–63) for SjD patients, and 29 years (IQR: 24–38) for HSs. Both SLE and SjD patients were older than HS patients, with a statistically significant difference (*p* < 0.001). Also, the erythrocyte sedimentation rate was statistically higher in both diseases than in HSs (*p* < 0.001). Regarding the treatment, the SLE patients showed higher exposure to glucocorticoids [*p* < 0.001, OR 24 (CI 95% 11.23–50.17)]. The remaining demographic and clinical characteristics of all study groups are presented in Table 1.

### 2.2. Genotypic and Alleles Frequencies of the rs61756766 [H159Y (475 C>T)] Polymorphism of TNFRSF13C Gene in Mexican Population

The HSs were in Hardy–Weinberg equilibrium for the rs61756766 polymorphism (X^2^ = 0.008, *p* = 0.976). The rs61756766 CC genotype was observed in 299 (99.7%) HSs, 295 (98.3%) patients with SLE, and in all patients with SjD. Conversely, the rs61756766 CT genotype was detected in only one (0.4%) HS and five (1.7%) SLE patients. None of the subjects carried the rs61756766 TT genotype. The C allele was the most prevalent in all study groups. When comparing the genotype and allele frequencies between SLE or SjD and HS, no statistically significant differences were found (*p* > 0.05; see Table 2).

### 2.3. Association of the rs61756766 [H159Y (475 C>T)] Polymorphism of TNFRSF13C Gen with Disease Activity in SLE and sBAFF Levels

The influence of this polymorphism on disease activity and chronicity in SLE patients was evaluated in this study. MexSLEDAI and SLICC-DI scores were comparable between carriers of the rs61756766 CC and rs61756766 CT genotypes (*p* > 0.05). Likewise, SLE patients with the rs61756766 CT genotype did not exhibit increased susceptibility to renal, mucocutaneous, or hematologic involvement, nor to active disease status, when compared with patients carrying the wild-type genotype (*p* > 0.05), see Table 3.

Conversely, SLE patients harboring the heterozygous CT genotype of the H159Y variant showed a non-statistically significant trend toward higher levels of soluble BAFF (sBAFF) compared to those with the wild-type genotype [2.88 (2.69–3.85) ng/mL (*n* = 192) vs. 1.97 (1.24–3.24) ng/mL (*n* = 5), respectively; *p* = 0.078]. Table 4 summarizes the clinical characteristics and sBAFF levels of these patients.

## 3. Discussion

SLE and SjD are autoimmune diseases in which inflammation is crucial to promoting tissue damage. One characteristic shared by both diseases is the presence of autoantibodies. B-cells are responsible for antibody production as they can differentiate into plasma cells. The expansion of autoreactive clones leads to the massive production of autoantibodies in certain autoimmune diseases, including SLE and SjD. BAFF and APRIL, through to BAFF-R, TACI, and BCMA receptors, are crucial for B-cell development, regulating their maturation during the transitional stage and differentiation into antibody-producing plasma cells. In the clinical setting, elevated levels of BAFF have been reported in the serum of patients with SLE and SjD [7]. This increase results in an imbalance between the negative selection of autoreactive clones, allowing their maturation, survival, and clonal expansion [11,16]. As a result, BAFF has become a therapeutic target for treating autoimmune diseases. Belimumab is a monoclonal antibody that targets BAFF and is FDA-approved to treat SLE patients, including severe manifestations such as lupus nephritis [17,18,19].

BAFF receptor (BAFF-R) is necessary for B-cell maturation in the transitional stage and is encoded by the *TNFRSF13C* gene. Few genetic variants have been explored regarding the expression of this receptor, where the H159Y (rs61756766) variant stands out. This single-nucleotide substitution (C>T) at position 475 of the third exon leads to an amino acid change from histidine to tyrosine in a region adjacent to the TRAF3 binding site in the cytoplasmic tail that leads to a hyperactivation of the receptor upon recognizing BAFF [12,20].

In this study, allelic and genotypic distribution frequencies of the H159Y variant (rs61756766) were similar between SLE patients and HS, while the variant was not found in SjD patients. These allele frequencies in the Mexican population are similar to the ones reported in the American population (C = 0.991, T = 0.009) (1000 Genomes Project—rs61756766 Allele Frequency, 2024, p. 1000 Genomes Project) and in the Latin American population (C = 0.989, T = 0.011) (Alpha Project—rs61756766 Allele Frequency, 2024, p. ALFA Project). These results contrast with studies in the European population, where an Italian cohort of Sjögren’s Disease patients showed a higher frequency (C = 0.952, T = 0.048), even exceeding frequencies reported in European populations by genotyping projects. The same case was for the Greek population, where they found a frequency of the heterozygous genotype of 3% in SLE patients (*n* = 145), 5% in patients with SjD (6.9%, *n* = 247), and 3% in patients with rheumatoid arthritis (*n* = 101) [15]. European cohorts typically exhibit more homogeneous ancestry and larger LD blocks, which can aid in detecting associations that are more difficult to identify in admixed populations such as the Mexican cohort. The Western Mexican population is genetically composed of European and Native American ancestry, at 64.9% and 30.8%, respectively [21]. Despite European ancestry, the Mexican population has a distribution similar to that reported in the Genomes project and Latin American reports; therefore, Native American ancestry could explain this finding.

Our study found no statistical association between the frequency of the H159Y variant (rs61756766) and the risk of developing SLE and its clinical manifestations or SjD in the study population. On the other hand, European studies have found that the presence of this variant is closely related to the development of lymphoma derived from SjD. In addition, a higher prevalence of the H159Y polymorphism was observed in European patients with multiple sclerosis, suggesting that increased BAFFR signaling could also contribute to the pathogenesis of the disease [14,15]. In another clinical setting, a recent study found that this variant is associated with susceptibility to developing severe clinical symptoms in patients in Campania (a region in southern Italy) infected with SARS-CoV-2 [13].

Interestingly, the SLE patient carriers of the H159Y variant (rs61756766) included in this study showed sBAFF levels above 2 ng/uL, which are comparable to the levels reported in the groups of SLE patients with a severe activity index and SjD patients, both diseases in the Mexican population [7,8]. In vitro studies have demonstrated that the presence of the H159Y variant in BAFF-R increases TRAF3 recruitment and NF-kB2 pathway activation [12,15]; consequently, this variant may induce BAFF-R hyperactivation, elevating sBAFF levels and promoting the survival of self-reactive B-lymphocytes in patients with SLE and the H159Y heterozygous genotype. A detailed analysis of the therapeutic regimen of the recruited patients with SLE revealed that all had two or more medications, with most receiving immunosuppressants and glucocorticoids (Table 3). Despite having more than a year since diagnosis, their treatment indicates that remission has not been achieved, and they continue to experience relapses, emphasizing the importance of BAFF as a cytokine involved in the disease activity.

Finally, B-cell ontogeny is regulated not only by BR3 and BAFF. APRIL, TACI, and BCMA are other components of the BAFF system that regulate the development of B-cells, and the soluble forms of the BAFF system receptors can be cleaved with few known physiological effects. Regarding BCMA, APRIL, and BAFF, Salazar-Camarena et al. explored the distribution of membrane BCMA (mBCMA), soluble BCMA (sBCAM), BAFF, and APRIL in SLE. Compared with HS and focused on BCMA, SLE patients have an aberrant expression of mBCMA with a higher concentration of sBCMA, mainly in active disease, proposing it as a biomarker of disease [22]. In addition to the clinical utility of BAFF and APRIL in SLE, Telitacicept is a novel dual inhibitor of BAFF and APRIL that has recently been evaluated for the treatment of autoimmune diseases such as SLE and rheumatoid arthritis [23]. All these findings highlight the importance of the BAFF system in the pathophysiology and treatment of autoimmune diseases, mainly in SLE.

From a clinical perspective, our findings do not support the routine screening for the H159Y (rs61756766) mutation in the gene encoding the BAFF-R receptor (TNFRSF13C) in all SLE or SjD patients. Although this variant could increase BAFF (sBAFF) levels in SLE patients, its presence does not associate with a more severe clinical phenotype or increased organ damage. However, the main endpoint of these studies is to enhance understanding of the role of the BAFF/BAFF-R axis in the pathophysiology of autoimmune diseases.

The main strengths of this work are the large sample size, which allowed us to perform a robust statistical analysis and determine that this variant does not confer risk or susceptibility for the development of SLE and SjD in the Mexican population. This study has several limitations, including its monocentric design and the narrow focus on a limited population from Western Mexico. Additionally, we observed a low frequency of the mutant allele, which does not rule out small effects from this polymorphic variant. Another limitation is the absence of functional assays using cells from individuals with the H159Y variant. Conducting these assays could provide insights into how B lymphocytes respond when activated through the BAFF/BAFF-R pathway. Understanding this response could clarify the potential effects of this variant in patients with SLE, SjD, or other conditions associated with BAFF/BAFF-R pathway activation.

## 4. Materials and Methods

### 4.1. Subjects

This study included a total of 710 Mexican participants, comprising 300 individuals diagnosed with SLE and 110 with primary SjD. All patients fulfilled the respective classification criteria: the 2012 Systemic Lupus International Collaborating Clinics (SLICC) criteria for SLE [24] or the 2016 American College of Rheumatology/European League Against Rheumatism (ACR/EULAR) criteria for primary Sjögren’s Syndrome (recently renamed Sjogren’s Disease) [25]. Additionally, 300 healthy subjects (HS) with no personal or family history of autoimmune diseases were included. To estimate the sample size, we use the formula for comparing proportions, based on the study by Jasek M. et al. 2016 [26]. The required sample size was 536.58 alleles, namely 268 subjects per group.

### 4.2. Clinical Assessments

SLE patients were clinically assessed using the Mexican Systemic Lupus Erythematosus Disease Activity Index (MexSLEDAI) and the Systemic Lupus International Collaborating Clinics/American College of Rheumatology Damage Index (SLICC-DI) [15,23]. In contrast, disease activity and damage in patients with SjD were evaluated using the Sjögren’s Syndrome Disease Activity Index (SSDAI) and the Sjögren’s Syndrome Disease Damage Index (SSDDI), respectively [27].

### 4.3. DNA Extraction and Genotyping

The genomic DNA (gDNA) was obtained from peripheral blood leukocytes using Miller’s technique [28]. The polymerase chain reaction (PCR)–restriction fragment length polymorphism (RFLP) technique was used for genotyping the rs61756766 (475C>T) polymorphism of the TNFRSF13C gene. The nucleotide sequence of the primers used was forward 5′CCT CCA GAG GAG TCT TCT AG3′ and reverse 5′TCC AAG CCC CTG GCT GGG3′. The Kompoti et al. conditions were used to obtain the PCR under the following protocol: initial denaturation cycle at 94 °C for 3 min; 30 cycles of denaturation for 30 s at 94 °C; annealing for 20 s at 57 °C; extension for 30 s at 72 °C; and final extension for 3 min at 72 °C [29].

The 323 bp PCR product corresponding to the rs61756766 (475C>T) polymorphism was digested using 0.5 units of the *MscI* restriction enzyme (New England BioLabs^®^, Ipswich, MA, USA) at 37 °C for 60 min. The resulting digestion fragments were as follows:

- C/C genotype: two bands of 181 bp and 142 bp;
- C/T: three bands of 323 bp, 181 bp, and 142 bp;
- T/T: a single band of 323 bp.

### 4.4. Serum BAFF Levels

The concentration of serum BAFF was quantified using a standardized sandwich enzyme-linked immunosorbent assay (ELISA) kit (DBLYS0B; R&D Systems, Minneapolis, MN, USA), according to the manufacturer’s instructions. A standard curve was constructed using serial dilutions of recombinant human BAFF (40 ng). The assay exhibited a sensitivity range of 1.01 to 6.44 pg/mL. Optical density was measured at 450 nm using a microplate reader (BioTek Instruments Inc., Winooski, VT, USA).

### 4.5. Statistical Analysis

The data were analyzed using SPSS v25 (IBM Corporation, Armonk, NY, USA) and GraphPad Prism v10.2.3 (GraphPad Software, San Diego, CA, USA) software. The variables were expressed in the median with interquartile range (IQR) and frequencies. To minimize the risk of genetic effects, the Hardy–Weinberg equation (p^2^ + 2pq + q^2^ = 1) was applied in HS to determine the expected allele frequencies and to report our population as being in equilibrium with the observed allele frequencies. Prior to conducting the statistical analysis, the Kolmogorov–Smirnov test was utilized, which obtained a nonparametric data distribution. Alleles and genotype frequencies were compared using the Chi-squared and Fisher’s exact tests. The comparison of soluble BAFF levels was calculated with a Mann–Whitney U test. A *p*-value < 0.05 was considered statistically significant.

## 5. Conclusions

Based on the frequencies found in this study, we can conclude that the H159Y variant does not confer risk for the development of SLE and SjD in the population of western Mexico, and the functional effect on BAFF-R could be related to the high levels of sBAFF in SLE patients. This added to the in vitro and clinical evidence in patients with diseases such as SLE, SjD, and SARS-CoV-2 shows that this is a variant worth studying in populations with a higher prevalence.

## Figures and Tables

**Table 1:** Demographic and clinical features of patients with SLE and SjD, as well as HSs.

Variable	SLE *(n* = 300)	SjD (*n* = 110)	HS (*n* = 300)
Age, years	34 (25–47)	56 (48–63)	29 (24–38)
Female gender, *n* (%)	274 (91.3)	110 (100)	237 (91.1)
Evolution of the disease, years	4 (1–9)	3 (1–8)	-
MexSLEDAI	2 (0–6)	-	-
Inactive (0–1), *n* (%)	118/280 (42.1)	-	-
Mild–moderate (2–5), *n* (%)	113/280 (40.3)	-	-
Severe (≥6), *n* (%)	49/280 (17.5)	-	-
SLICC-ACR Damage Index	0 (0–1)	-	-
Patients with SLE damage, *n* (%)	97/281 (34.5)	-	-
SSDAI	-	2 (0–3)	-
Patients with active disease (SSDAI > 5), *n* (%)	-	6/104 (5.8)	
SSDDI	-	1 (0–2)	-
Patients with SjD damage, *n* (%)	-	76/104 (73.1)	
Autoantibody profile			
Antinuclear antibodies (ANAs), *n* (%)	300/300 (100)	50/71 (70.4)	-
ANAs title	640 (320–1280)	320 (160–800)	-
Staining pattern			
Nuclear speckled, *n* (%)	117/217 (53.9)	44/50 (88)	
Nuclear homogeneous, *n* (%)	95/217 (43.7)	1/50 (2)	-
Anti-dsDNA+, *n* (%)	131/249 (52.6)	-	-
Anti-RNP+, *n* (%)	45/117 (38.4)	-	-
Anti-SSA/Ro+, *n* (%)	33/114 (28.9)	24/104 (23.0)	-
RF+, *n* (%)	39/169 (23.0)	39/97 (40.2)	-
Anti-Sm+, *n* (%)	30/133 (22.5)	-	-
Anti-SSB/La+, *n* (%)	15/122 (12.2)	16/104 (15.3)	-
Clinical domains			
Hematological, *n* (%)	119/261 (45.5)	6/104 (5.8)	-
Mucocutaneous, *n* (%)	87/261 (33.3)	0	-
Renal, *n* (%)	60/262 (22.9)	0	-
Musculoskeletal, *n* (%)	49/261 (18.7)	42/104 (40.4)	-
Neurological, *n* (%)	11/264 (4.1)	0	
Pulmonary/Serositis, *n* (%)	9/261 (3.4)	0	-
Glandular involvement, *n* (%)	0	8/104 (7.7)	
Constitutional involvement, *n* (%)	0	58/104 (55.8)	-
Vascular involvement, *n* (%)	16/283 (5.7)	3/104 (2.9)	-
Laboratory variables			
ESR (mm/h)	30.5 (16–47)	26 (14–34)	13 (8–21)
Treatment			
Prednisone use, *n* (%)	189/283 (66.7)	8/104 (7.6)	-
Prednisone dosage	10 (5–20)	3.75 (1.8–5)	-
Prednisone > 15 mg/day, *n* (%)	56/283 (19.7)	0/104 (0.0)	-
Antimalarial, *n* (%)	169/283 (59.7)	-	-
Azathioprine, *n* (%)	142/283 (52.2)	-	-
Methotrexate, *n* (%)	55/283 (19.4)	22/104 (21.1)	-
Mycophenolate mofetil, *n* (%)	21/283 (7.4)	-	-
Cyclophosphamide, *n* (%)	25/283 (8.8)	-	-

Data are shown in median with IQR, frequencies, and percentages, according to the case. Abbreviations: SLE, Systemic Lupus Erythematosus; SJD, Sjögren’s Disease; MexSLEDAI, Mexican Systemic Lupus Erythematosus Disease Activity Index; SLICC-ACR, Systemic Lupus International Collaborating Clinics/American College of Rheumatology; SSDAI, Sjögren’s Syndrome Disease Activity Index; SSDDI, Sjögren’s Syndrome Disease Damage Index; ANAs, antinuclear antibodies; Anti-dsDNA, double-stranded DNA; RF, rheumatoid factor; IQR: interquartile range.

**Table 2:** Frequencies of genotypes and alleles of the TNFRSF13C gene polymorphisms in SLE and SjD patients.

rs61756766 (475 C>T) Genotypic and Allelic Frequencies							
Genotype	HS *n* = 300	SLE *n* = 300	*p-*Value	OR (CI 95%)	SjD *n* = 167	*p-*Value	OR (CI 95%)
CC	299 (99.7%)	295 (98.3%)	-	1	109 (100%)	-	1
CT	1 (0.3%)	5 (1.7%)	0.1	5.0 (0.6–59.9)	0 (0.0%)	0.5	0.0 (0.0–24.5)
TT	0 (0.0%)	0	-	-	0 (0.0%)	-	-
Alleles							
C	595 (99.2)	599 (99.8)	-	1	218 (100%)	-	1
T	5 (0.8)	1 (0.2)	0.22	5.03 (0.6–43.2)	-	-	-

The *p*-value and OR (CI 95%) represent the SLE and SjD frequencies compared with HC; the *p*-value was obtained through the Fisher test or Chi-square test, according to the case. Abbreviations: HS, healthy subjects; SLE, Systemic Lupus Erythematosus; SjD, Sjögren’s Disease; OR, odds ratio; and CI, confidence interval.

**Table 3:** Comparison of main clinical domains according to genotypes of the TNFRSF13C gene polymorphisms in SLE patients.

rs61756766 (475 C>T) in SLE			
Domain	CC (%)	CT (%)	*p* Value [OR (IC 95%)]
Kidney	58/257 (22.57)	2/5 (40)	0.323 [2.29 (0.39–11.38)]
Hematological	115/255 (45.1)	4/5 (80)	0.182 [4.87 (0.78–59.96)]
Mucocutaneus	86/170 (50.6)	1/5 (20)	0.667 [0.49 (0.04–3.053)]

Data are shown in frequencies and percentages; the *p*-value was obtained through the Fisher test or Chi-square test, according to the case. Abbreviations: HS, healthy subjects; SLE, Systemic Lupus Erythematosus; OR, odds ratio; and CI, confidence interval.

**Table 4:** Clinical features and sBAFF levels of SLE patients with rs61756766 CT genotype of TNFRSF13C gene.

Case	rs61756766 (475 C>T)	Age	sBAFF	Evolution of Disease	Disease Activity	PDN	CLQ	AZA
	Genotype	Years	ng/mL	Years	MexSLEDAI	mg/Day	mg/Day	mg/Day
1	CT	31	2.88	3	1	5	150	100
2	CT	32	2.74	1	7	10	150	100
3	CT	37	2.88	2	1	15	-	150
4	CT	29	4.82	5	1	30	150	150
5	CT	33	2.64	9	2	-	75	-

Abbreviations: sBAFF: soluble BAFF; BAFF, B-cell activation factor; SLE, Systemic Lupus Erythematosus; PDN, prednisone; CLQ, chloroquine; and AZA, azathioprine.

## Data Availability

The original contributions presented in this study are included in the article. Further inquiries can be directed to the corresponding author.
